# Retraction Note: Micronized calcium carbonate to enhance water-based drilling fluid properties

**DOI:** 10.1038/s41598-024-82508-2

**Published:** 2024-12-10

**Authors:** Salem Basfar, Salaheldin Elkatatny

**Affiliations:** Department of Petroleum Engineering, King Fahd University of Petroleum and Minerals, 31261 Dhahran, Saudi Arabia

Retraction of: *Scientific Reports* 10.1038/s41598-023-45776-y, published online 25 October 2023

The Editors have retracted this Article.

After publication, concerns were raised regarding the veracity of the following data presented in this work,

- the X-ray diffraction (XRD) pattern labeled Conventional CaCO_3_ shown in Figure 4;
- the statistical analysis of the effect of calcium carbonate on the density of the water-based drilling fluid, shown in Figure 5.

The Authors provided raw data on request from the Editors. However, the Editors found that the original XRD pattern is different from that presented in the Article. The Editors therefore no longer have confidence in the research presented in this work.

None of the Authors have responded to correspondence from the Editors about this retraction.

